# How the metabolic machinery of maximally exercising muscle fails

**DOI:** 10.1113/JP279670

**Published:** 2020-04-04

**Authors:** Graham J Kemp

**Affiliations:** ^1^ Department of Musculoskeletal Biology and Liverpool Magnetic Resonance Imaging Centre (LiMRIC) University of Liverpool UK

**Keywords:** mitochondria, muscle metabolism, nuclear magnetic resonance, ^31^P MRS

Four decades after it was first applied to skeletal muscle, phosphorus magnetic resonance spectroscopy (^31^P MRS) remains a valuable noninvasive window into metabolism *in vivo*. Bartlett *et al*. (2020) use ^31^P MRS to investigate a physiological puzzle: why skeletal muscle O_2_ consumption is higher than expected during maximal exercise (Roston *et al*. 1987).

Muscle's ability to match ATP turnover to mechanical output, still not fully understood, also provides a useful way to probe the system. By quantifying key metabolites (inorganic phosphate, phosphocreatine (PCr) and ATP directly, [H^+^] and [ADP] indirectly), dynamically during exercise and recovery, ^31^P MRS can provide information on several aspects of muscle metabolism: ATP use by the contractile apparatus and non‐contractile processes such as the Ca^2+^‐ and Na^+^‐K^+^‐ATPases; the balancing production of ATP by oxidative phosphorylation (dominant in low‐intensity and sustained exercise) and anaerobic glycogenolysis (important as workload increases); the creatine kinase equilibrium which buffers temporary mismatch of ATP supply and use; and the cellular acid‐base physiology which mitigates and eventually reverses the pH change accompanying anaerobic glycogenolysis to lactate (Kemp, 2015). ^31^P MRS has significant technical complications and limitations (Meyerspeer *et al*. 2020): absolute quantification is not straightforward, many interesting metabolites are inaccessible, and analysis often involves physiological assumptions and mathematical models. Other techniques have different strengths and limitations: muscle biopsy gives access to a wider range of metabolites, but time‐series measurements during exercise are very challenging, especially in clinical studies. So too are direct arteriovenous cannulation studies of muscle transmembrane fluxes (e.g. O_2_ in, lactate out). The noninvasive (whole‐body) measurement of pulmonary respiration offers ready but indirect access to muscle oxidative metabolism. Alone or complemented by these techniques, ^31^P MRS has a place in the *in vivo* muscle physiology toolkit.

The phenomenon this paper investigates was originally identified by studies measuring pulmonary respiration: during high‐intensity exercise (i.e. above the lactate threshold), steady‐state oxygen uptake rates are greater than expected (Roston *et al*. 1987). This phenomenon is related to increased recruitment of Type II (fast twitch, less oxidative) fibres in the working muscle, but the underlying physiology is debated: it might reflect an increasing ATP cost of contraction (so more O_2_ is consumed because more ATP is needed), or else progressive mitochondrial uncoupling (so more O_2_ is needed to generate the required ATP). This might seem easily resolved by measuring all the relevant fluxes, but practical obstacles are formidable. Several studies have nevertheless attempted to distinguish these possibilities by combining techniques, as the paper notes.

Bartlett *et al*. did not choose a multimodal approach, but used one of the most conceptually straightforward ^31^P MRS analyses: the initial post‐exercise PCr resynthesis rate as a measure of the end‐exercise suprabasal oxidative ATP synthesis rate. A practical disadvantage is that this requires exercise to be stopped, but it is straightforward to repeat exercise at various durations to build up a time‐course. The key finding is that the oxidative ATP synthesis rate decreases during later exercise. This raises two questions.

First, does this reflect a genuine shortfall in ATP supply, or is less ATP made because less is needed? ^31^P MRS offers a simple way to address ATP cost: the initial PCr depletion rate measures total ATP turnover at the start of exercise, but this obviously cannot be repeated to generate a time‐course. Instead, the authors exploit cellular acid‐base physiology to estimate non‐oxidative ATP synthesis rates throughout the exercise period, to show that contractile efficiency does not change. On a technical note, it is perhaps unsatisfactory that several different analytical models and methods are still advocated, requiring assumptions about buffer capacity and mitochondrial feedback regulation, for example, which have some experimental support, but are not definitively established (Kemp *et al*. 2015). To address this the authors used several alternative analysis methods, which turn out to have little effect on the conclusions.

The key finding is thus that ATP cost does not change, at least in this protocol, so mitochondrial ATP synthesis is indeed progressively falling short of what is needed. The second question, then, is why? Here again ^31^P MRS can help. It is well established that the first‐order rate constant of post‐exercise PCr resynthesis is a measure of what might be called ‘effective mitochondrial capacity’. This concept depends on the notion of feedback control of oxidative ATP synthesis via the creatine kinase equilibrium, and can be thought of as an estimate of the hypothetical rate of ATP synthesis given maximal values of feedback signals like [ADP] (Kemp *et al*. 2015). The finding is that this decreases with exercise. Between muscles of different fibre composition or trained and untrained subjects, for example, this quantity reflects the number and functionality of mitochondria (Kemp *et al*. 2015). When it changes during exercise, it clearly reflects acute changes in mitochondrial function. These experiments cannot prove a mechanism, but the finding is compatible with a mitochondrial uncoupling mechanism.

In summary, the authors have exploited the ability of ^31^P MRS to study metabolic events in muscle exercising at high intensity, and thrown some useful light on an important physiological phenomenon, the over‐consumption of O_2_ by maximally exercising muscle. The results strongly implicate a progressive impairment of mitochondrial function, and the authors suggest an uncoupling‐based mechanism, which points the way to the studies which will be needed to pin this down.

## Additional information

### Competing interests

The author has no conflicts of interest to report.

### Funding

GJK's work is supported by the Medical Research Council UK and Versus Arthritis as part of the Medical Research Council Versus Arthritis Centre for Integrated research into Musculoskeletal Ageing (CIMA) (MR/K006312/1).
